# A Special Report on 2019 International Planning Competition and a Comprehensive Analysis of Its Results

**DOI:** 10.3389/fonc.2020.571644

**Published:** 2020-12-04

**Authors:** Jiayun Chen, Jianrong Dai, Ahmad Nobah, Sen Bai, Nan Bi, Youqun Lai, Minghui Li, Yuan Tian, Xuetao Wang, Qi Fu, Bin Liang, Tao Zhang, Wenlong Xia, Yuan Xu, Wenting Ren, Xuena Yan, Ji Zhu, Deqi Chen, Jiming Yang

**Affiliations:** ^1^ Department of Radiation Oncology, National Cancer Center/National Clinical Research Center for Cancer/Cancer Hospital, Chinese Academy of Medical Sciences and Peking Union Medical College, Beijing, China; ^2^ Radiation Physics Section, Biomedical Physics Department, King Faisal Specialist Hospital & Research Centre, Riyadh, Saudi Arabia; ^3^ Department of Radiation Oncology, West China Hospital, Sichuan University, Chengdu, China; ^4^ Department of Radiation Oncology, Fujian Medical University Xiamen Humanity Hospital, Xiamen, China; ^5^ Department of Radiotherapy and Chemotherapy, Ningbo First Hospital, Ningbo, China

**Keywords:** radiotherapy, lung cancer, planning competition, multicenter, simultaneous integrated boost

## Abstract

**Purpose:**

The aim of this work is to introduce the 2019 International Planning Competition and to analyze its results.

**Methods and materials:**

A locally advanced non-small cell lung cancer (LA-NSCLC) case using the simultaneous integrated boost approach was selected. The plan quality was evaluated by using a ranking system in accordance with practice guidelines. Planners used their clinical Treatment Planning System (TPS) to generate the best possible plan along with a survey, designed to obtain medical physics aspects information. We investigated the quality of the large population of plans designed by worldwide planners using different planning and delivery systems. The correlations of plan quality with relevant planner characteristics (work experience, department scale, and competition experience) and with technological parameters (TPS and modality) were examined.

**Results:**

The number of the qualified plans was 287 with a wide range of scores (38.61–97.99). The scores showed statistically significant differences by the following factors: 1) department scale: the mean score (89.76 ± 8.36) for planners from the departments treating >2,000 patients annually was the highest of all; 2) competition experience: the mean score for the 107 planners with previous competition experience was 88.92 ± 9.59, statistically significantly from first-time participants (*p* = .001); 3) techniques: the mean scores for planners using VMAT (89.18 ± 6.43) and TOMO (90.62 ± 7.60) were higher than those using IMRT (82.28 ± 12.47), with statistical differences (*p* <.001). The plan scores were negligibly correlated with the planner’s years of work experience or the type of TPS used. Regression analysis demonstrated that plan score was associated with dosimetric objectives that were difficult to achieve, which is generally consistent with a clinical practice evaluation. However, 51.2% of the planners abandoned the difficult component of total lung receiving a dose of 5 Gy in their plan design to achieve the optimal plan.

**Conclusion:**

The 2019 international planning competition was carried out successfully, and its results were analyzed. Plan quality was not correlated with work experiences or the TPS used, but it was correlated with department scale, modality, and competition experience. These findings differed from those reported in previous studies.

## Introduction

Radiotherapy (RT) is suitable for approximately 50% of all cancer patients (1). Treatment planning is a major component of RT and is usually performed by trained planners. These planners design an arrangement of radiation sources to meet the clinical goals. Key questions involve how to achieve a high-quality treatment plan and correlating plan quality with relevant characteristics of the planner and with technological parameters. With the aims of improving the quality of RT professionals’ work and sharing best practice knowledge among participants all over the world, the Chinese Society of Medical Physics (CSMP) and Radiation Knowledge (RK) jointly sponsored the remote 2019 international planning competition (hereafter, “the competition”). This 2019 competition was the first remote planning competition event organized through collaboration between two large organizations in medical physics. The CSMP was established in 1981 and subsequently joined the International Organization for Medical Physics in 1986. The CSMP led the competition, and RK provided related websites and resources.

Participants could join the 2019 competition freely after registering. In contrast to the previous eight competitions organized by RK, in 2019, the CSMP selected a team of experts to contribute to the competition. An expert committee system was thus established for the first time for the purpose of ensuring the fairness and impartiality of the competition. The mission of the expert committee included case selection, beta testing of the test plan, scoring metric development, eligibility determination for submitted plans, and ranking of the top 20 winners. As a baseline the plans generated must be clinically treatable. The committee hoped to help medical physicists and medical dosimetrists improve their clinical skills, especially in terms of treatment planning techniques.

Previous competitions have been held annually, organized by various organizations and with different formats. The results of only two of these competitions have been published in scientific journals. First, in early 2011, Nelms et al (2). studied the outcome of a competition of 125 plans for treating a prostate cancer case, concluding that the plan quality was not statistically related to technological parameters (treatment planning system [TPS], modality, and plan complexity) or planner characteristics (years of experience, confidence, certification, and education). Second, in 2018, Moustakis et al (3). reported on a competitive multiplatform benchmark treatment planning challenge for spinal radiosurgery with 12 participating centers. There are two limitations regarding these previous studies that should be addressed. First, prostate cancer plans are not particularly challenging in clinical practice; therefore, plans formulated for the treatment of such cases may not adequately reflect the skill of the planners or distinguish the advantages of specific modalities. Second, the treatment planning techniques used 10 years ago may not represent the “optimal” trade-off between 1) dose conformity and sparing normal tissue (NT) and 2) treatment efficiency (4). Over more than 10 years of industrial technology development, linear accelerator characteristics have advanced greatly in dose delivery systems such as multi-leaf collimator design (5–8), and there has been great improvement in the accuracy of dose computation in the dose calculation algorithms available in commercial products (9–12). Therefore, it is crucial to examine a new round of competition representing the complex clinical treatment plans produced today. Furthermore, it is high time for an update of the investigation of the quality of a large population of treatment plans.

Lung cancer is the leading cause of cancer death worldwide for both men and women (13). Definitive RT is the standard treatment for locally advanced non-small-cell lung cancer (LA-NSCLC), but the outcome following treatment remains poor because of excessive radiation-induced toxicity (14, 15). The simultaneous integrated boost (SIB) approach can simultaneously deliver an intense dose to the gross tumor and a reduced dose to the subclinical area, resulting in improved normal-tissue sparing and treatment tolerance. The clinical efficacy and safety of SIB-IMRT/VMAT have recently been demonstrated for LA-NSCLC (16–18), and this approach has been widely implemented in clinical practice. The SIB approach for LA-NSCLC is quite complex in clinical use. Unfortunately, no previous studies have determined the optimal SIB plan for treating LA-NSCLC, which platforms and methods can achieve such a plan, or the relevant characteristics of planners.

For the above mentioned reasons, the 2019 competition’s expert committee selected a clinical LA-NSCLC case with an SIB approach. Using the 2019 competition database of a large population of treatment plans, we investigated the quality of plans designed by planners worldwide who used different planning and delivery systems. In this article, we report the correlations of plan quality with the relevant characteristics of the planner and with technological parameters.

## Materials and Methods

### Competition Process and Characteristics of the Participating Planners

The expert committee announced the competition 1 month ahead of the start date. At this time, participants in previous competitions received an invitation for the 2019 competition and were asked to share the competition poster with their colleagues and others in the same occupation. After registering for the competition, participants could receive notifications regarding the competition progress to avoid missing submission deadlines.

As a part of the registration process, all participants were required to complete a 13-question survey. This survey collected information on the participant location, job title, previous planning competition participation, work experience, and total department workload. These data were collected to assess the composition of the participants and to enable the analysis of relationship between the high plan score and planner characteristics as well as the technological parameters.

The participating treatment planners were trained clinicians (physicians, dosimetrists, and medical physicists), students, residents, and sometimes technicians employed by TPS vendors. Hereafter, we refer to these individuals collectively as “the planners.”

### Competition Timeline and Awards

The competition’s start and end dates are as follows:

(1) Opening of registration: June 29, 2019
(2) Start date: July 15, 2019
(3) Plan submission deadline: August 25, 2019
(4) Plan evaluation: September 10, 2019
(5) Top 20 planners contacted: September 20, 2019
(6) Awards announcement: October 1, 2019

The top 20 participants received a certificate acknowledging their ranking in this international competition, and the top five received an award. In case of a tie, an expert team evaluated the plans and then voted to determine the ranking of the two plans. The expert team evaluated the plans in terms of their rationality and deliverability for clinical use. The plan evaluation scores were calculated to three decimal places. The CSMP provided awards and certificates for the top-ranked planners who met all competition requirements. RK provided certificates for participants who completed the competition requirements and submitted their files.

### Case Description

As mentioned above, an LA-NSCLC case was used for the 2019 competition. The patient had already undergone treatment in the Department of Radiation Oncology at Cancer Hospital, Chinese Academy of Medical Science (CAMS). A 58-year-old man was admitted to the hospital due to a cough persisting for 4 months. Chest computed tomography (CT) showed a mass in the right lower lung with metastasis in mediastinal lymph nodes. Pathological examination found squamous cell carcinoma. Brain magnetic resonance imaging was unremarkable. A positron emission tomography scan showed a mass with fluorodeoxyglucose uptake in the right lower lung. Mediastinal (2R, 3A, 4R, 7) and right hilar lymph nodes metastases were found. Other observations were unremarkable. The patient had a history of smoking. The diagnosis was squamous cell carcinoma in the right lower lung with lymph nodes metastasis stage (7th AJCC) T3N2M0, stage IIIA.

### Immobilization and Simulation

The patient was immobilized in the supine position with a thermoplastic custom-made mask (including a head, neck, and shoulder mask and a chest mask). A 4D-CT scan with contrast enhancement was obtained using an 850 mm big bore CT scanner (Philips Healthcare, Andover, MA, USA) with 512 × 512 pixels at 5-mm intervals in the axial helical mode. The scanned regions extended from the laryngeal prominence to the bottom of the L2 vertebral body. The patient underwent a respiration-correlated 4D‐CT scan using the Varian RPM System (Version 1.7). The 4D‐CT images were reconstructed in 10 equally spaced phase bins using an Advantage 4D Workstation (Philips Healthcare, Andover, MA, USA), where the maximum-intensity projection images and average images were generated. The average CT was used as the planning image. The 4D, maximum-intensity projection and average images were imported into the TPS and co-registered for target delineation.

### Target Volume and Organs at Risk Delineation

The Radiotherapy and Oncology Group guidelines (19) and International Commission on Radiation Units 83 report (20) served as references for the delineation of target volume and organs at risk (OARs). The gross tumor volume (GTV) was contoured based on the 10 phases of 4D-CT images, labeled as GTV00-GTV90 respectively. GTV00-GTV90 involved the primary lesions and positive lymph nodes, which were defined as those with a short-axis diameter of at least 1 cm on each phase of the CT images or of less than 1 cm but high fluorodeoxyglucose (FDG) uptake on images. IGTV is the fusion of GTV00 to GTV90. GTVnd was metastatic lymph nodes in the right hilar and mediastinum (2R, 3A, 4R, 7) based on the positron emission tomography results.

The clinical target volume (CTV) was generated by expanding GTV by 0.6–0.8 cm, covering the involved hilum and mediastinal nodal stations. CTV included the following:

(1) IGTV + 6 mm;
(2) GTVnd + 5 mm; and
(3) area of the right hilar and mediastinum (2R, 3A, 4R, 7).

**Figure 1:**
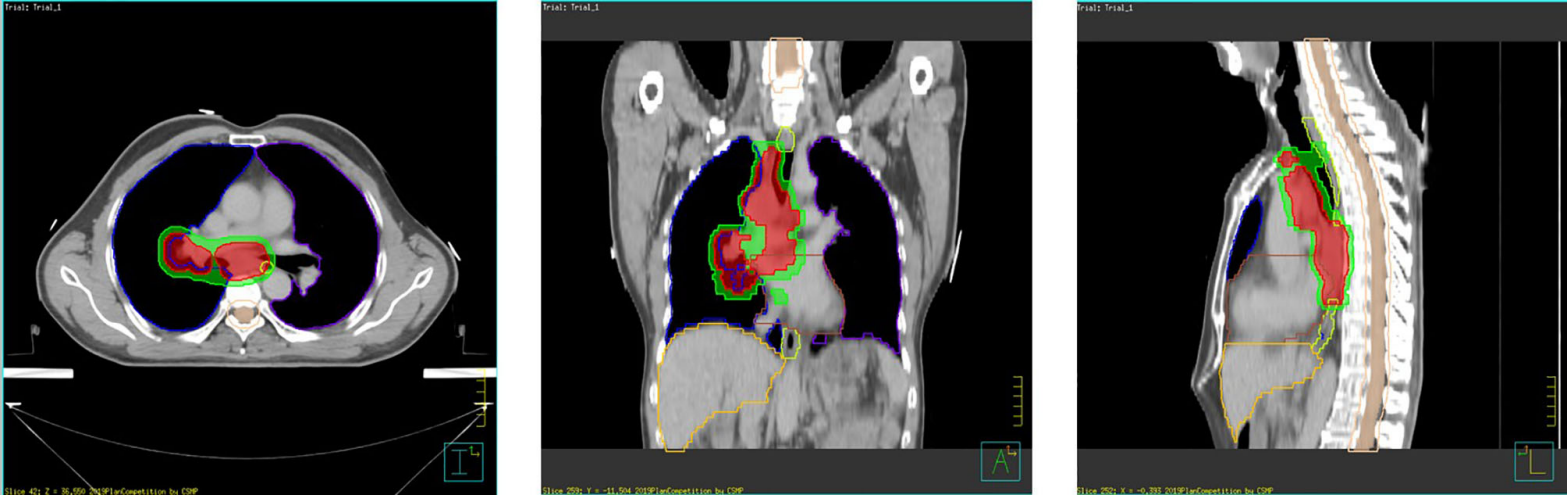
Case presentation—axial, sagittal, and coronal views.

The planning target volume (PTV) was created by a uniform expansion of 0.5 cm surrounding the CTV. The planning gross tumor volume (PGTV) was the GTV expanded by 0.5 cm. The lungs, heart, esophagus, and spinal cord were contoured as the dose constraint for OARs. **Figure 1** shows the anatomy planes and contour reconstructions through the cross-section of PGTV and PTV.

### Prescribed Dose and Dose Constraints

The prescribed dose was 50.4 Gy in 28 fractions to the PTV and 60.2 Gy in 28 fractions to the PGTV. The dose should be prescribed to cover ≥95% of the target volumes (PTV/PGTV). The maximum dose should be less than 110% of the prescribed dose. The dose constraints for OARs were determined with reference to the values summarized in **Table 1**. These were based on a phase 2 clinical trial (21) and the clinical experience of CAMS (16). OARs dose volume constraints were based on our department guidelines (22), which were stricter than the guidelines of the National Comprehensive Cancer Network (23) and other institutions. There were two reasons for applying our department’s guidelines: First, the rate of pneumonitis has been found to be relatively high in Asian patients (data from the PACIFIC 2nd analysis) (24). Second, the volume of heart receiving 40 Gy (V_40 Gy_) has been found to be significantly associated with overall survival (data from the RTOG0617 2nd analysis) (25).

**Table 1:** Dosimetric criteria.

**Structure Name**	**Query**	**Metric Type**	**Target**	**Tolerance**	**Score**
PGTV	V60.2 Gy[%]	min	95.00	90.00	9.00
PTV	V50.4 Gy[%]	min	95.00	92.00	7.00
PGTV	CI60.2 Gy	min	0.85	0.65	3.00
PTV	CI50.4 Gy	min	0.75	0.55	3.00
PGTV	HI60.2 Gy	max	0.20	0.50	2.00
PTV-PGTV0.2	HI60.2 Gy	max	0.30	0.60	3.00
NT	Mean [Gy]	max	6.00	12.00	3.00
RVR	V50.4 Gy[%]	max	0.10	1.00	2.00
Total Lung	Mean [Gy]	max	16.00	20.00	6.00
Total Lung	V5 Gy[%]	max	55.00	75.00	5.00
Total Lung	V20 Gy[%]	max	25.00	35.00	12.00
Total Lung	V30 Gy[%]	max	18.00	25.00	8.00
Cord	V40 Gy[cc]	max	0.01	1.00	9.00
Cord PRV	V45 Gy[cc]	max	0.01	1.00	8.00
Heart	V30 Gy[%]	max	35.00	45.00	5.00
Heart	V40 Gy[%]	max	25.00	30.00	5.00
Esophagus	V50 Gy[%]	max	30.00	50.00	3.00
Liver	V30 Gy[%]	max	1.00	10.00	3.00
Liver	V5 Gy[%]	max	10.00	50.00	2.00
PGTV	Global Max Dose	bool	YES	0.00	2.00
				**Max Score:**	**100.00**

The baseline OARs dose volume constraints were as follows:

(1) The total lung (lungs – GTV) volumes receiving more than 20 Gy (V_20_) and 30 Gy (V_30_) were limited to <30 and <20%, respectively. V_5_ was limited to 55%.
(2) The mean dose to the total lungs should optimally be <17 Gy and should not exceed 20 Gy.
(3) Spinal cord PRV: <45 Gy
(4) Heart: V_30_ < 40%, V_40_ < 30%
(5) Esophagus: V_50_ < 40–50%
(6) Liver: V_30_ < 30%

### Planning Objectives and Patient Dataset

The main goal of the treatment plan was the “optimal” trade-off between 1) dose conformity and sparing NT and 2) treatment efficiency. The planning objectives were as follows:

(1) To control the dose level of two targets (PGTV and PTV)
(2) To achieve high conformity for two targets (PGTV and PTV) and homogeneity of PTV−PGTV0.2 (definition provided in the next section)
(3) To spare the OARs surrounding the targets. The NT should receive a reasonably low irradiated dose according to the “as low as reasonably achievable” principle.
(4) To improve planning skills by sharing the methods used by the competition winners.

Patient data were anonymized and made available for download from the official competition webpage as DICOM3 images (CT) and a DICOM RT structure set (contours).

### Quality Ranking of the Plans

The participating planners were provided with the exact treatment plan objectives and plan scoring system software used to rank the quality of submitted plans. The dosimetric criteria for the plans were defined and discussed by the expert committee of seven professional planners and one radiation oncologist. There were 20 dosimetric objectives, with a maximum total score of 100, as presented in **Table 1**. The 100-point score comprised the following components:

(1) Target coverage and dose conformity: 22 points
(2) Target homogeneity index: 5 points
(3) OARs: 71 points
(4) Other: 2 points

The expert committee generated four structures in addition to the targets and OARs. The planning OAR volume (PRV) for the spinal cord, denoted as “spinal cord PRV,” was created by adding a 5-mm margin. A supporting structure for the NT was created for the area surrounding the PTV range, and the general low-dose loading in the scanned area was evaluated. NT represents the external contour of the patient’s body minus the PTV (6). PTV−PGTV0.2 is the PTV extracted from the PGTV expanded by 0.2 cm. The remaining volume at risk (RVR), recommended by the International Commission on Radiation Units 83 report (26), was defined as the difference between the volume enclosed inside the external contour (body) and the defined structures (OARs and targets).

The conformation index represents the dose fit of the target relative to the volume covered by the prescribed isodose lines, which were defined following Paddick (27). The homogeneity index was defined following Herman et al (26). V_n Gy_ (%) was the percentage of the organ volume receiving ≥n Gy (5). D_Vcc_ and D_mean_ were the near-maximum absorbed dose (where V was a small fractional volume) and average absorbed dose delivered to each OAR, respectively.

All submitted plans were required to be clinically deliverable. The basic principles of the plans were described as follows:

(1) Only one isocenter was allowed in each submitted plan.
(2) The use of non-coplanar fields/arcs was not allowed. No more than 12 beams for IMRT and no more than four arcs for VMAT.
(3) The dose calculation grid size was required to be <3 mm.
(4) The pencil beam dose calculation algorithm was not allowed.
(5) Use of the dose heterogeneity option was required for the dose calculation.
(6) Changing the multi-leaf collimator/jaws transmission factors of the submitted plan would disqualify the planner from the competition.
(7) The submitted plans had to be clinically treatable (reasonable beam-on time):
  - IMRT plan: approximately 20 min
  - VMAT plan: approximately 10 min
  - Tomotherapy plan: approximately 30 min
(8) The plan scoring system software used was PyPlanScoring (created by RK; hereafter referred to as “the scoring system”). The scoring system was compatible with plans generated through most TPS and evaluated plan quality using defined dosimetric criteria. To avoid variance in the contours, the scoring system used the original structures provided to the planners for plan evaluation rather than the planners’ contours.
(9) Adding optimization structures like rings or virtual structures was allowed.
(10) Converting the resolution of some OARs to high resolution was allowed.
(11) Assigning an HU number to the structures was not allowed.

The planners had 6 weeks to design and fine-tune their plans. Only essential treatment plan data were collected from all planners in the form of a DICOM RT plan and dose files at the end of the competition. The files were to be saved as zip files and named using a standard format (Final_Scores_TPS_Techinque_Email address.zip).

### Statistical Analysis

Statistical analyses were performed using SPSS, Version 23.0 (IBM Corp, Armonk, NY, USA). The Shapiro–Wilk test was applied to verify that the data were normally distributed, and a one-way ANOVA (analysis of variance) was conducted. Bonferroni’s *post-hoc* multiple comparison test was used for pairwise comparisons. In the case of non-normally distributed values or variance heterogeneity, the non-parametric Kruskal–Wallis test was used to compare groups of data. A significance level of *p* <.05 was used for all tests.

## Results

**Figure 2:**
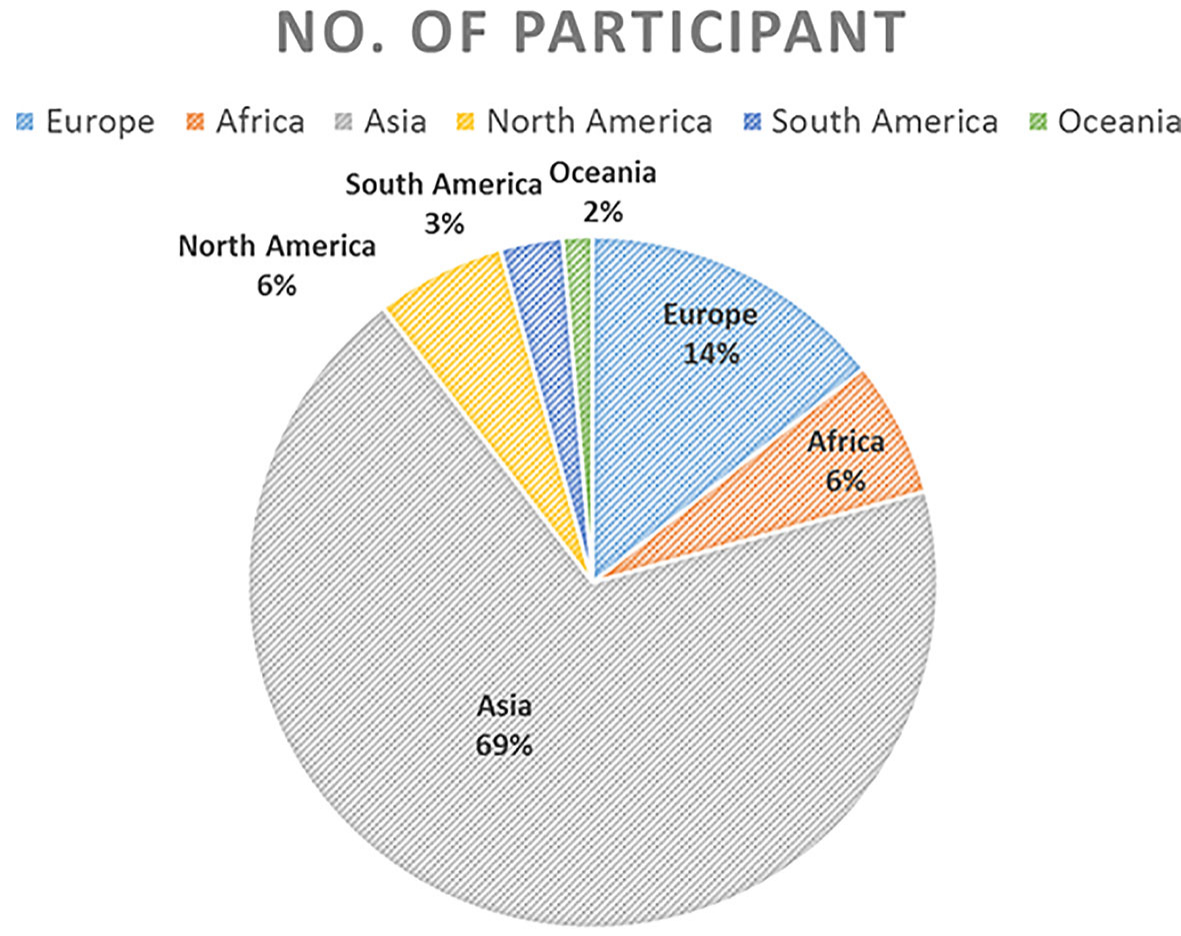
Number of participating planners by continent and overall.

In total, 1,301 participants from 76 countries all over the world registered for the competition and downloaded the CT DICOM and RT structure, as well as the scoring system (**Figure 2**). The largest number of participants was based in Asia (*n* = 896) because of the large population of this region. This was followed by European planners (*n* = 187), who made up 14.37% of the total participants. A total of 383 plans were received, and 287 of these were regarded as qualified for this study. Data cleaning and consolidation principles were as follows:

(1) Every plan was evaluated by the scoring system. Volunteer staff members double-checked the score with the file labeled score. If the labeled score differed from the evaluated score, a second volunteer staff member would be involved. The expert committee decided the final scores for all plans.
(2) The plan could not be evaluated if the RT dose or RT plan file was missing. Plans missing either of these files were removed from the database.
(3) There were multiple entries from several individual planners. In these cases, the highest scoring plan for each planner was retained, and the planner’s other plans were removed to avoid potential bias in the study.

**Figure 3:**
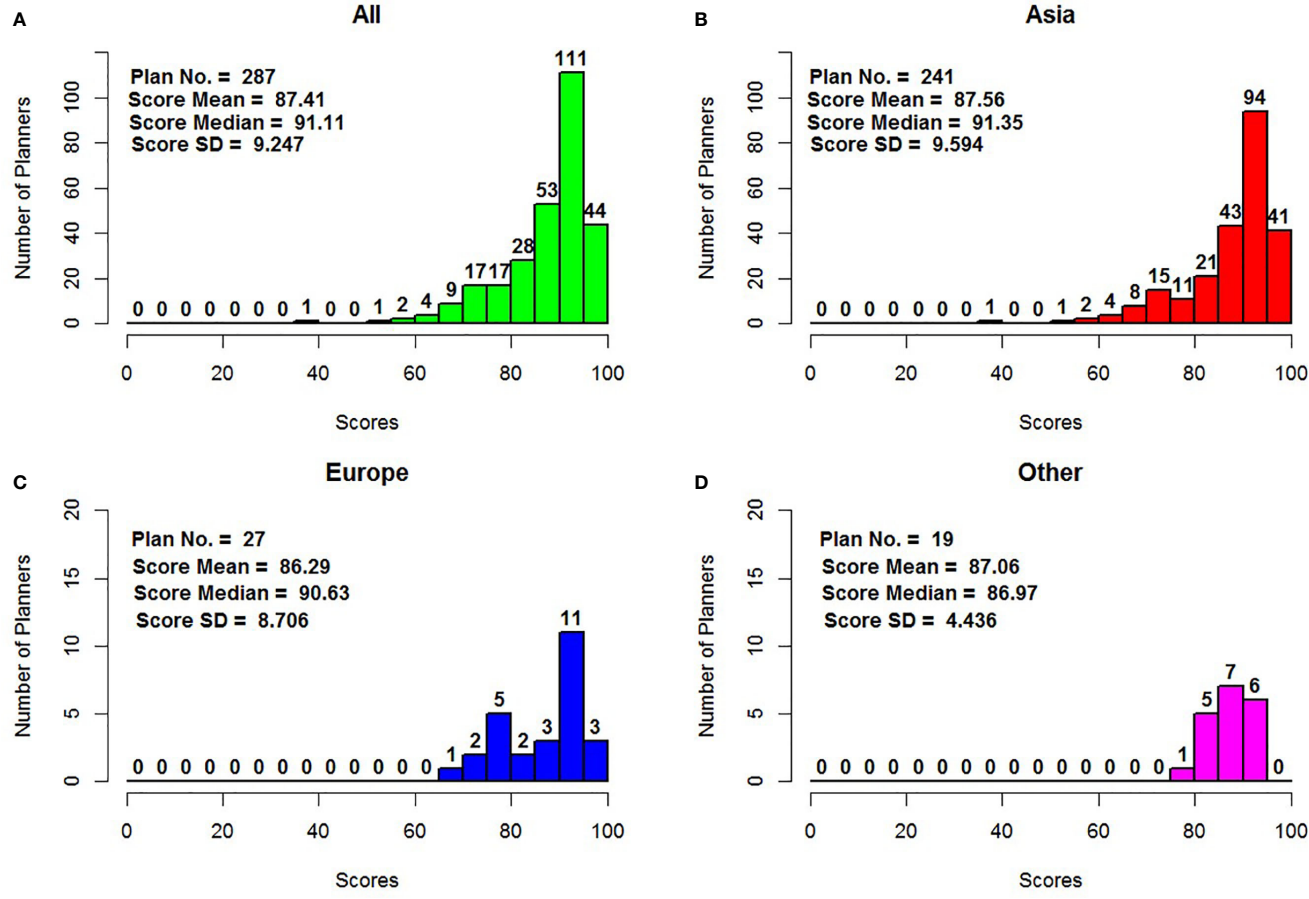
Plan score distribution by continent and overall **(A)** overall **(B)** for Asia **(C)** for Europe, and **(D)** for other continents including North America, South America, and Oceania.

The plan score distribution for all eligible plans is shown in **Figure 3**. There was a wide range of scores (38.61–97.99), with a mean of 87.41, a standard deviation of 9.25, and a median of 91.11. In total, there were 241 eligible plans from Asia in this study, with a mean of 87.56 ± 9.60 and a median 91.35, which was slightly higher than the overall mean/median plan score. European planners contributed 27 plans, with a mean of 86.29 ± 8.71. The planners from other continents (including North America, South America, Oceania, and Africa) submitted 19 plans, with a mean score of 87.06 ± 4.44. The mean scores of the plans submitted by European planners and those from other continents were slightly lower than the mean score for all plans. Over 54% of all plan scores were higher than 90, including 135 plans from planners in Asia.

**Figure 4:**
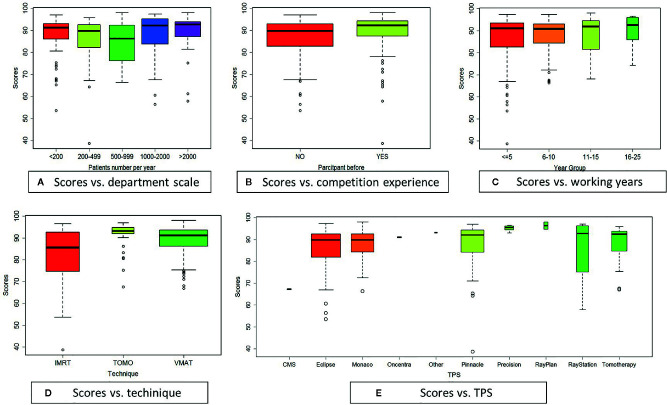
Boxplots graphically presenting correlations with plan score: **(A)** department scale, **(B)** competition experiment, **(C)** working year, **(D)** technique, **(E)** TPS.

The plan scores are presented in **Figure 4**, which also shows the correlations of the plan score with planner characteristics (department scale, competition experience, and work experience) and with the treatment plan platforms (technique and TPS). The boxplot widths are proportional to the square root of the sample size.

The scores showed statistically significant differences by department scale, as is depicted in **Figure 4A**. The department scale reflected the number of patients treated per year in a planner’s department. The number of patients treated annually was categorized as <200, 200–499, 500–999, 1,000–2,000, and >2,000. The planners were evenly distributed across these department scale categories. Planners from departments that treated >2,000 patients per year had the highest mean score (89.76 ± 8.36), as shown in **Table 2**. The second- (88.84 ± 9.12) and third- (87.26 ± 9.26) highest mean scores were found among planners from departments that treated 1,000–2,000 and <200 patients each year, respectively.

**Table 2:** Mean plan score by department scale.

**Department Scale**	**Score: Mean ± SD**	**Planners No.**	**p-values**
<200	87.258 ± 9.259	60	–
200–499	85.412 ± 10.540	46	<0.025^e^
500–999	85.165 ± 8.564	58	<0.02^d,e^
1,000–2,000	88.839 ± 9.118	64	<0.02^c,^
>2,000	89.760 ± 8.355	59	<0.025^b,c^

A two-sided p-value <0.05 was considered statistically significant. Significant differences were found when comparing each category with the following numbers of patients treated annually in the planner’s department: ^a^<200, ^b^200–499, ^c^500–999, ^d^1,000–2,000, ^e^>2,000. “-” indicates that no significant differences were found.

The distributions of plan scores by previous competition experience are shown in **Figure 4B**. The mean score was 88.92 ± 9.59 for the 107 planners who had previously participated and 86.51 ± 8.95 for the 180 planners who were participating in the competition for the first time. Thus, planners with competition experience had a higher mean score, and the independent-sample Kruskal–Wallis test showed this difference to be statistically significant (*p* = .001).

The plan scores were negligibly correlated (*p* = .487) with planner’s years of work experience. **Figure 4C** shows a mean score of 86.48 ± 10.60 for the 128 planners with less than 5 years of work experience, 88.05 ± 7.73 for the 110 planners with 6–10 years of work experience, 87.65 ± 9.03 for the 33 planners with 11–15 years of work experience, and 89.89 ± 7.43 for the 16 planners with >16 years of work experience. The mean plan score by years of work experience category is shown in **Table 3**. Over 82.9% of the planners had worked for <10 years.

**Table 3:** Mean plan score by years of work experience.

Working years	Score: Mean ± SD	Planner No.
≤5	86.476 ± 10.595	128
6–10	88.054 ± 7.728	110
11–15	87.648 ± 9.025	33
16–25	89.891 ± 7.427	16

Plan quality was statistically significantly associated with the technique used: the mean scores for both VMAT (89.18 ± 6.43, 171 planners) and TOMO (90.62 ± 7.60, 35 planners) were higher than that for IMRT (82.28 ± 12.47, 81 planners), as presented in **Table 4** and **Figure 4D**. The differences between VMAT and IMRT and between TOMO and IMRT were statistically significant, as shown by the independent-sample Kruskal–Wallis Test (*p* <.001). VMAT and TOMO, instead of IMRT, have become the most common forms used in most RT centers, and the number of patients treated using these techniques is increasing each year. Planners using these two novel techniques were more skillful at implementing advanced clinical techniques. These findings differ from those of Nelms et al (2).

**Table 4:** Mean plan score by technique used.

Techniques	Score: Mean ± SD	Planner No.	p-values
IMRT	82.283 ± 12.468	81	<0.001^b,c^
TOMO	90.618 ± 7.594	35	<0.001^a^
VMAT	89.175 ± 6.434	171	<0.001^a^

A two-sided p-value <0.05 was considered statistically significant. Significant differences were found when comparing each category with the following techniques: ^a^IMRT, ^b^TOMO, ^c^VMAT.

The plan scores were negligibly correlated with most TPS, as shown in **Figure 4E** and **Table 5**. TPS from eight commercial TPS vendors were included in the dataset: CMS XiO (Elekta, Stockholm, Sweden), Eclipse (Varian Medical Systems, Palo Alto, CA, USA), Monaco (Elekta AB, Stockholm, Sweden), Oncentra (Elekta AB, Stockholm, Sweden), Pinnacle (Philips, Madison, WI, USA), Precision (Accuracy, Sunnyvale, CA, USA), RayPlan and RayStation (RaySearch Medical Laboratories AB, Stockholm, Sweden), and Tomotherapy (Tomotherapy, Madison, WI, USA). A total of 103 planners (35.88%) used Eclipse (mean score: 86.23 ± 9.01), 62 used Pinnacle (mean score: 87.84 ± 10.25), and 61 used Monaco (mean score: 87.99 ± 6.70). Over 78.75% of the participating planners used one of these three TPS modalities as their competition TPS. There were no statistically significant differences in plan score between these three TPS modalities (*p* >.05). There was a significant difference in plan score between the CMS and Precision TPS (*p* <.02), but this finding should be interpreted with caution because of the small numbers of planners using these TPS (CMS: *n* = 2, Precision: *n* = 9).

**Table 5:** Mean plan score by TPS modality.

TPS modaliy	Score: Mean ± SD	Planner No.	p-values
CMS	67.218 ± 0.403	2	<0.02^g^
Eclipse	86.294 ± 9.005	103	<0.01^g^
Monaco	87.994 ± 6.700	61	<0.02^g^
Oncentra	90.971 ± 0.357	2	–
Other	93.073	1	–
Pinnacle	87.843 ± 1 0.248	62	–
Precision	95.062 ± 1.340	9	<0.02^a,b,c^
RayPlan	96.325 ± 2.352	2	–
RayStation	85.427 ± 13.910	17	–
Tomotherapy	88.333 ± 8.946	28	–

A two-sided p-value <0.05 was considered statistically significant. Significant differences were found when comparing each category with the following TPS modalities: ^a^CMS, ^b^Eclipse, ^c^Monaco, ^d^Oncentra, ^e^other, ^f^Pinnacle, ^g^Precision, ^h^RayPlan, ^i^RayStation, ^j^Tomotherapy. “-” indicates that no significant differences were found.

## Discussion

A challenging lung cancer case was chosen for the 2019 Radiotherapy Plan Competition: the SIB approach for LA-NSCLC is a very complex clinical case. The present study reported the results of a well-constructed multi-center planning competition that included planners from around the world, as well as an analysis of differences in plan score by multiple variables. We found plan quality to be negligibly correlated with work experience and TPS, which is consistent with Nelms et al.’s findings in early 2011 (2). Years of work experience do not appear to represent planners’ skill level. Although the planners participating in the competition used various TPS algorithms (12) and previous work has reported differences in the dose calculation accuracy of different TPS (28), our findings suggest that plan quality is not statistically significantly associated with the TPS used.

We found that plan quality was correlated with department scale, techniques used, and competition experience. These findings demonstrate that several factors other than general planner skill affect plan quality, which differs from previous studies conducted by Nelms et al. in early 2011 (2) and by Moustakis et al. in 2018 (3).

Planners from departments that treated >2000 patients per year had the highest mean score likely reflecting the relatively high opportunity of planners in large departments to deal with complex clinical cases. Notably, the planners in the smallest departments achieved good plan quality. These planners may have had more planning time to dig in and focus on the particular competition case, because of lighter clinical routine. Planners have not a rush on the planning deadline. Planners working in departments treating 500–999 patients per year exhibited a dip in plan scores. Importantly, the collected data only indicate the total patient workload for a department, and the ratio of planners to patients was not available. If the ratio of planners to patients is slightly lower in departments treating 500–999 patients per year than in higher-volume institutions, the planners in the former type of departments may have been too busy to carefully review and optimize their plans. This may explain why the mean score for planners in such departments was lower than the mean scores of planners in departments treating smaller or larger volumes of patients.

In our study, the scores of VMAT plans were higher than those of IMRT plans. This finding is consistent with Xhaferllari et al.’s (4) study, which indicated that VMAT is dosimetrically advantageous in treating early-stage NSCLC with SABR, compared with fixed-beam IMRT. The VMAT and TOMO plans were comparable in our study, which is inconsistent with Xhaferllari et al.’s (4) conclusion the VMAT outperformed TOMO on all measured parameters. However, we noticed a significant increase in contralateral V_5 Gy_ for all TOMO plans, and we observed that the planning constraints were not set the same for the different techniques in Xhaferllari et al.’s (4) study. This may be because of a lack of experience with the TOMO techniques, resulting in insufficient optimization of the plans. In the competition examined in our study, the planners made a strong effort with their plans and tried to achieve the objectives. This situation may have reduced the circumstances where technical advantages are not fully utilized.

Our study was the first to report a correlation between plan quality and competition experience. Planners who have previously participated in a competition may be more familiar with the competition process and rules, compared with first-time participants. Planners with previous competition experience could thus save time and focus more on the planning, perhaps even avoiding low-level mistakes. Indeed, planners with previous competition experience were found to obtain better scores. The top performer had participated in previous competitions, and he believed that this experience improved his skills and knowledge in treatment planning techniques and optimization algorithms. In the 2019 competition, this planner first analyzed the possibility of achieving the required objective for each target and OAR and then classified the targets and OARs as a function of their achievement difficulty degree based on an objective function. He then investigated the interaction between the OARs objectives and adjusted his approach accordingly.

In this competition, the competition expert committee selected 20 components into as the dosimetric criteria. It was essential to use methodology like this dosimetric criteria mechanism to ensure that all planners were planning to meet specific and exact objectives (2), as in previous competitions. Besides, we considered that the dosimetric goals of five OARs components and two targets components were hard difficult to satisfy: 1) Targets: PGTV conformation index (CI) (PTV–PGTV), homogeneity index (HI); 2) OARs: Total Lung V_5_, V_20_, and D_mean_; RVR V_50.4_; NT D_mean_. The regression analysis is a set of statistical methods used for the estimation of relationships between a dependent variable and one or more independent variables. It can be utilized to assess the strength of the relationship between components. Using regression analysis, we found that exhibited plan score was associated with both targets and OARs. The regression formulas for the targets and OARs are as follows:

(1) $$\begin{array}{l} \text{Targets: Final Score}\\ =71.96+7.18\times \text{PGTV}\;\text{CI}\\ -0.91\times (\text{PTV}-\text{PGTV})\text{HI} \end{array}$$

(2) $$\text{OARs: Final Score}=71.25+1.53\times \text{Total Lung}\;{\text{V}}_{20}+5.76\times \text{RVR}\;{\text{V}}_{50.4}+2.63\times \text{Total Lung}\;{\text{D}}_{mean}-9.06\times \text{NT}\;{\text{D}}_{\text{mean}}$$

The regression analysis was consistent with clinical practice characteristic of the competition case. The components above are definitely hard to achieve objectives on clinical practice. The expert committee tried to distinguish the optimal plan with single specifically dosimetric criteria. The wide range distribution of plan scores reflects the success of this strategy. As the clinical routine is busy for most planners, it is more suitable to determine the ranking by one match. The planner will give up the competition if they need to do more than one shot. However, one match strategy needs more works, plan betas and thoughtful discussion of the expert committee.

Meanwhile, one bias was notice and found by planners in this dosimetric metrics. V_5_ of total lung was the hardest objective to meet and a clinical evaluating indicator. Radiation-induced pneumonitis (RP) is the most common dose-limiting complication of LA-NSCLC treated by thoracic radiotherapy. A recent study indicated that total lung V_5_ was one of the dosimetric parameters associated with the occurrence of radiation-induced pneumonitis (29). However, the score on this component was zero for 147 submitted plans, indicating that 51.2% of all participating planners abandoned this component in their plan design to achieve a “better” plan. The maximum score for total lung receiving 5 Gy was only five points. One could abandon this five score and try to achieve the dosimetric criteria of the other 19 components. This strategy was easier than trying to fulfill all 20 components. Another weakness of this study is that the plans are self-selected and therefore not necessarily representative of “real-life” plans actually implemented for patients. The competition metrics for assessing submitted plans should avoid manipulations of this kind in the future by introducing aspects of clinical routine processes. The components related to clinical evaluation indicators should be required meet a minimum limit. Plans that do not meet this minimum limit should regard as failed plans.

Finally, an undirected limitation of this study was that the only participants who thought they achieved the “optimal” plan in their mind submitted their plans to the competition. We found that about 30% of the planners who registered for the competition ultimately submitted a plan. This rate is has been seldom reported by the competition organizations. Attempts were made to contact planners who registered for the competition and to encourage them to share their plans; registered participants were contacted by email at five different time points before the competition deadline. Most of the planners who registered for the competition downloaded the documentation and even attempted to create an optimized plan. However, many ultimately did not submit a plan. Because planners participated in the competition and submitted their plans voluntarily, when they did not believe that their score would be high enough, they may have been unwilling to submit their plans. Although the competition committee promised not to release any personal information, such as individual planners’ names and employers, along with their scores, the planners may have felt that the only planners with high scores should share their plans. An additional possibility is that planners who did not believe their plans were of high quality refrained from submitting them as they did not believe they would be competitive. Some planners may also have downloaded the case documents simply because they were interested in the case and wanted to attempt to formulate a plan, without intending to submit their plan. Increasing the submitted plan rate could be a useful goal for the organizers of future plan competitions.

## Conclusions

The 2019 Radiotherapy Plan Competition successfully hosted and analyzed competition results. This study was innovative because it comprehensively studied a plan competition in medical physics and identified several new factors that affected plan quality, using a large population of qualified plans submitted by planners around the world.

Plan quality was not correlated with work experience or TPS, but was correlated with department scale, techniques, and competition experience. Our findings demonstrated that plan quality is affected by several factors besides general planner skill, which differs from the results of previous studies. Advanced treatment systems including linear accelerators, techniques, and TPS modalities currently play an important role in RT plan development.

## Data Availability Statement

The datasets generated and/or analyzed during the current study are not publicly available due to data security requirement of CSMP and Cancer Hospital, Chinese Academy of Medical Sciences. Requests to access the datasets should be directed to JC, grace_chenjy@163.com.

## Ethics Statement

The studies involving human participants were reviewed and approved by This study was carried out in accordance with the declaration of Helsinki and approved with exemption from informed consent by the independent ethics committee of Ethics Committee of National Cancer Center/Cancer Hospital, Chinese Academy of Medical Sciences and Peking Union Medical College (Approval No.: 20/194-2390). Written informed consent for participation was not required for this study in accordance with the national legislation and the institutional requirements.

## Author Contributions

All authors discussed and conceived of the study design. JC was involved in competition organization and one of the expert committee, performed data analysis, and drafted the manuscript. AN provided the related websites resources and helped to collect the data. The expert team committee members are JD, SB, NB, YL, ML, YT, XW, and JC. The mission of the expert team committee included the case selection, plan beta, score metric development, the eligibility of submitted plans, and winners ranking. TZ contoured the targets and organs at risk of the case, and NB approved it. QF was responsible for the communication with the participants by the competition official emails and answering the frequent questions from the participants. WX and YX drafted two packages of instruction documents for the competition. WR and JC advertised the competition. XY, JZ, DC, and JC evaluated all plans. JD led the competition Organization and guided the study preparation of the manuscript. BL participated in discussions about the data analysis and provided meaningful suggestions. All authors contributed to the article and approved the submitted version.

## Conflict of Interest

The authors declare that the research was conducted in the absence of any commercial or financial relationships that could be construed as a potential conflict of interest.
